# Lignans in Knotwood of Norway Spruce: Localisation with Soft X-ray Microscopy and Scanning Transmission Electron Microscopy with Energy Dispersive X-ray Spectroscopy

**DOI:** 10.3390/molecules25132997

**Published:** 2020-06-30

**Authors:** Tuomas Mansikkala, Minna Patanen, Anna Kärkönen, Risto Korpinen, Andrey Pranovich, Takuji Ohigashi, Sufal Swaraj, Jani Seitsonen, Janne Ruokolainen, Marko Huttula, Pekka Saranpää, Riikka Piispanen

**Affiliations:** 1 Nano and Molecular Systems Research Unit, Faculty of Science, University of Oulu, P.O. Box 8000, FI-90014 Oulu, Finland; leo.mansikkala@oulu.fi (T.M.); marko.huttula@oulu.fi (M.H.); 2 Biocenter Oulu, P.O. Box 5000, University of Oulu, FI-90014 Oulu, Finland; 3 Production Systems, Natural Resources Institute Finland, Latokartanonkaari 9, FI-00790 Helsinki, Finland; anna.karkonen@luke.fi (A.K.); risto.korpinen@luke.fi (R.K.); pekka.saranpaa@luke.fi (P.S.); 4 Viikki Plant Science Centre, Department of Agricultural Sciences, University of Helsinki, FI-00014 Helsinki, Finland; 5 Wood and Paper Chemistry Research Group, Laboratory of Natural Materials Technology, Åbo Akademi University, Porthansgatan 3, FI-20500 Turku, Finland; andrey.pranovich@abo.fi; 6 UVSOR Facility, Institute for Molecular Science, 38 Nishigo-naka, Myodaiji, Okazaki, Aichi 444-8585, Japan; ohigashi@ims.ac.jp; 7 SOLEIL Synchrotron, L’Orme des Merisiers, Saint-Aubin, P.O. Box 48, CEDEX, FR-91192 Gif-Sur-Yvette, France; sufal.swaraj@synchrotron-soleil.fr; 8 Nanomicroscopy Center, Department of Applied Physics, Aalto University, P.O. Box 15100, FI-00076 Aalto, Finland; jani.seitsonen@aalto.fi (J.S.); janne.ruokolainen@aalto.fi (J.R.)

**Keywords:** *Picea abies*, knotwood, extractives, hydroxymatairesinol, lignin, resin-embedding, cryosectioning, STXM, STEM-EDS, osmium tetroxide

## Abstract

Lignans are bioactive compounds that are especially abundant in the Norway spruce (*Picea abies* L. Karst.) knotwood. By combining a variety of chromatographic, spectroscopic and imaging techniques, we were able to quantify, qualify and localise the easily extractable lignans in the xylem tissue. The knotwood samples contained 15 different lignans according to the gas chromatography-mass spectrometry analysis. They comprised 16% of the knotwood dry weight and 82% of the acetone extract. The main lignans were found to be hydroxymatairesinols HMR1 and HMR2. Cryosectioned and resin-embedded ultrathin sections of the knotwood were analysed with scanning transmission X-ray microscopy (STXM). Cryosectioning was found to retain only lignan residues inside the cell lumina. In the resin-embedded samples, lignan was interpreted to be unevenly distributed inside the cell lumina, and partially confined in deposits which were either readily present in the lumina or formed when OsO_4_ used in staining reacted with the lignans. Furthermore, the multi-technique characterisation enabled us to obtain information on the chemical composition of the structural components of knotwood. A simple spectral analysis of the STXM data gave consistent results with the gas chromatographic methods about the relative amounts of cell wall components (lignin and polysaccharides). The STXM analysis also indicated that a torus of a bordered pit contained aromatic compounds, possibly lignin.

## 1. Introduction

Norway spruce (*Picea abies* L. Karst.) knots contain lignans that comprise 2–24% of the knotwood dry weight, whereas in the sapwood and heartwood of the stem, the lignan content is negligible. Thus, the knots are a rich source of extractable lignans [1,2,3]. Norway spruce lignans are mainly composed of hydroxymatairesinol (HMR). The spruce knots contain up to 17% of HMR [4]. Lignans are optically active dimers synthesised from coniferyl alcohol, and HMR can be isolated as a mixture of two diastereomers: (7*R*,8*R*,8’*R*)-(−)-7-*allo*-hydroxymatairesinol (minor isomer) and (7*S*,8*R*,8’*R*)-(−)-7-hydroxymatairesinol (major isomer) [4]. Their biosynthesis is related to lignin, one of the main structural cell wall components of wood. Lignans are optically active because they are composed of only one enantiomer, or of two enantiomers with either one being dominant [5]. Lignin, on the other hand, is a randomly structured polymer with no optical activity.

Lignans are strong antioxidants with anti-tumour and wound healing effects, as well as hormonal activity. They have been used as components in the cosmetics, chemical and drug industries, and as constituent for Norway spruce resin salve used to heal wounds. The biological activity of lignans has been widely studied [4,6,7]. Lignans have been modified in the presence of nanometal (palladium) under acidic conditions [8,9,10]. The versatility of lignan molecules in their suitability for different processes and their functional properties have been frequently studied, but the properties of lignans in their native environment in the Norway spruce knotwood are not well known. Obtaining knowledge of the localisation of lignans in trees and within the xylem is essential to understanding their biosynthesis and role in wood protection and determining their further applications.

Axially along the tree stem, the lignan concentration is highest in knots located near the base of a living crown [3]. Based on our pilot study with confocal Raman IR microscopy, lignan molecules are hypothesised to be packed in specific structures in the knotwood cells [11]. However, the strong autofluorescence of lignin complicated the detailed structural analysis. We have demonstrated that a novel spectromicroscopy technique, scanning transmission X-ray microscopy (STXM) in a soft X-ray range, can be used to study the structure of wood cells, and to locate lignan regions in the Norway spruce knotwood cells [12]. While we were able to identify high contrast regions with the spectral fingerprints of lignans, the analysis was intricated by the fact that the samples were prepared using conventional protocols for transmission electron microscopy (TEM), i.e., embedded in resin and stained with OsO_4_. In the present study, we systematically extended the work by studying both resin-embedded and cryosectioned knotwood cells with the STXM technique, and also by using scanning transmission electron microscopy with high-angle annular dark-field imaging coupled with energy dispersive X-ray spectroscopy (STEM-HAADF-EDS, or STEM-EDS in short). Additionally, OsO_4_ binding to HMR was assayed to show the location of lignans in the Norway spruce STEM-EDS images.

TEM is a standard tool in sub-micrometer imaging, and it can be coupled with detection of X-ray photons emitted from the sample (STEM-EDS) or with an electron energy loss spectroscopy (EELS) analysis of the transmitted electrons. STEM-EDS and EELS can thus provide elemental and chemical maps of the sample, respectively. Compared to synchrotron-radiation-based STXM, TEM is more accessible, but has limited energy resolution in the EELS mode. Electron bombardment in TEM induces more significant radiation damage to the sample, and usually soft matter samples require special preparation, such as embedding in a matrix, sectioning, staining and carbon coating [13]. However, TEM can reach a sub-nm spatial resolution compared to the few tens of nm obtained in STXM. Previously, STEM-EDX and EELS have been used to study the ultrastructure and elemental composition of Norway spruce cells [14,15,16]. A quantitative analysis of K and Ca in different cell types of Norway spruce was conducted using TEM-EDS [14], and EELS was used to study the penetration of a partly methylated hydroxymethyl melamine resin into lignified cell walls [15]. STXM has also been used to study a wide variety of plant samples. Owing to the chemical sensitivity of the technique, the studies have been concentrated on mapping different biopolymers in oak (*Quercus* sp.) [17], lentil cells (*Lens culinaris*) [18], plant fossils (*Metasequoia milleri, Asteroxylon mackiei*) [19] and xylem cells in different trees [20]. STXM was combined with time-of-flight secondary ion mass spectrometry (TOF-SIMS) in a study where the effects of lignin-modifying and polysaccharide-degrading enzymes in aspen (*Populus tremuloides*) were evaluated [21].

The purpose of the present work was to develop methods to localise native lignans in the tissue of Norway spruce knotwood, and to gain novel knowledge about the molecular structure of wood, and determine how/if wood extractives are interacting with cell wall macromolecules. The aims were: (i) using model compounds as references, to differentiate lignin and lignan regions within Norway spruce knotwood cells with STXM and STEM-EDS; (ii) to gain insight into the possible chemical bonding of lignans within the cells; (iii) using STEM-EDS, to qualify and quantify the distribution of osmium (Os) in order to verify the preferential reaction of OsO_4_ with lignan deposits observed by STXM inside the cell lumina, and test the reactivity between HMR and OsO_4_; and (iv) to evaluate the feasibility and performance of STXM and STEM-EDS microscopy in the chemical imaging of Norway spruce xylem.

## 2. Results and Discussion

### 2.1. Structural Knotwood Compounds

In order to localise lignans in situ in Norway spruce knotwood xylem cells, concentrations of structural components and extractives in knotwood were analysed with GC-MS to facilitate the analysis process with STXM and to compare quantitative results from both methods. The carbohydrate composition of the (cryosectioned) knotwood sample used for STXM was determined by acid hydrolysis and acid methanolysis and the results are shown in Table 1. In the spruce knotwood, cellulose dominated among the other structural cell wall constituents, i.e., heteropolysaccharides and lignin (Table 1). In general, cellulose is the most abundant natural polymer, which typically amounts to 40–50% in bulk wood [22]. However, spruce knotwood contained substantially less cellulose than spruce stem wood, that is 37% in the knotwood vs. 46–47% in the spruce heartwood (HW) and the sapwood (SW), respectively [23].

Non-cellulosic polymeric carbohydrates, known as hemicelluloses and pectins, are the next large group of plant constituents and structural compounds in the wood cell wall. They are a complex mixture of different heteropolysaccharides built up with pentose and hexose sugar units, including uronic acids (Table 1). Their total content in the knotwood was similar to that in the spruce stem wood [23].

Similar to spruce stem wood, galactoglucomannan (GGM) is the principal hemicellulose in the spruce knotwood. However, it is important to note that the galactose content in the knotwood was apparently higher than that in the spruce SW/HW wood (Table 1) [23,24]. The doubled amount of galactose in the knotwood could be due to the substantial amount of galactan. According to Timell, a high amount of galactan in spruce wood is typical for the compression wood [25].

Xylan is another hemicellulose in spruce; it contains uronic acids in the side chains and thus carries an anionic charge. There was a similar xylan content in both the spruce stem wood and the corresponding knotwood (Table 1) [23]. Based on the molar ratio between xylose (a backbone sugar unit) and 4-*O*-Me-glucuronic acid (a side chain sugar unit), it can be supposed that xylan in the knotwood is less branched than that in the spruce stem wood tissues (0.8 vs. 1.2 4-*O*-Me-glucuronic acid units per 10 xylose units, respectively [24]).

Pectin in the secondary wood usually exists in a small quantity. It is a highly charged polysaccharide due to the large amount of galacturonic acid units, which are, however, methyl esterified to a high degree in the native wood [26]. Based on the amount of galacturonic acid detected (Table 1), pectin content in the knotwood was found to be higher than in the spruce stem wood (0.17–0.2%) [24].

Lignin is the second most abundant compound after cellulose in the spruce wood. In contrast to lignans, lignin is a structural compound of the cell walls, and contributes to the mechanical strength of wood tissues. It is an amorphous and polyphenolic substance, which mostly comprises guaiacyl (G) type phenylpropane units in stem wood. In compression wood, notable quantities of p-hydroxyphenyl (H) units can be present.

The proportion of lignin in the pre-extracted spruce knotwood was 29.7% by weight (29.4% Klason and 0.3% acid soluble lignins), which exhibited a bit higher value compared to that in stem wood. However, lignin content in spruce wood varies substantially from tree to tree, in different morphological parts of the same tree and between the cell wall layers. Even in mature, healthy, straight and knot-free spruce stem wood, lignin content varies between 25.9% and 28.9% of dry wood [22,25,27,28]. According to Hägglund and Larsson (1937) [29], the lignin content in the Norway spruce knotwood was 33.0%, which is a median value between 28.6% and 38.8% of lignin in stem wood and compression wood, respectively [25]. In our experiment, the knotwood was sampled close to the pith and above the centre of the knot, where compression wood is not present.

### 2.2. Extractives in the Knotwood

The extractive content of the Norway spruce knotwood sample that was also used for STXM (cryosectioned sample) was analysed by GC-GC/MS. Extractives obtained with acetone constituted approximately 20% of the wood dry weight (Table 2). The acetone extract contained lignans (81.8% of the extract), and out of the lignans ca. 59% and 22.5% were composed of HMR and other lignans, respectively (Table 3). The extract also contained sesqui- and dilignans (15.1%), fatty acids (0.32%) and dehydroabietic acid (0.08%) as minor components (Table 3).

### 2.3. X-ray Absorption Spectra of the Acetone Extract and of Selected Reference Compounds

**Figure 1:**
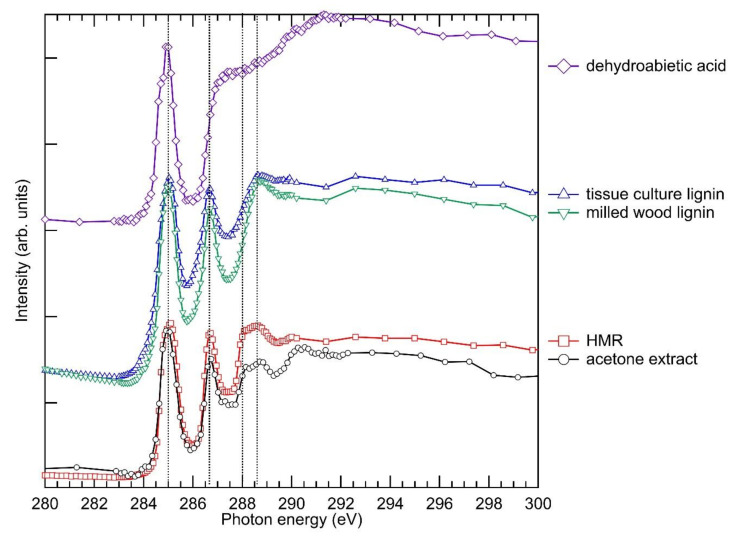
C 1s X-ray absorption spectra of the acetone extract of the knotwood (black line with circles), of hydroxymatairesinol (HMR, red line with squares), of the tissue culture lignin (blue line with triangles), of the milled wood lignin (green line with triangles) and of dehydroabietic acid (violet line with diamonds). The spectra of HMR and the tissue culture lignin have been adopted from Huttula et al. [12].

The localisation of the lignans using STXM was based on their specific spectral fingerprints in the X-ray absorption spectrum. In order to disentangle the different chemical components in the STXM measurement, C 1s X-ray absorption spectra (XAS) of some isolated and model compounds were measured for a reference (Figure 1). The corresponding comparison of O 1s XAS is presented in the supplementary material (Figure S1). An especially interesting region is the 285–287 eV, which contains two sharp peaks originating from the C1s→π* transition in the aromatic ring, with carbon and hydrogen substitution (aryl-C,H, 285 eV) and O substitution (aryl-O, 286.7 eV) [19]. This fingerprint region separates the lignin and lignan from the polysaccharide components of the wood cell walls [12,18,19]. The spectrum of the acetone extract was very similar to that of the HMR, the main difference being the change in the relative intensities of the spectral features. This observation is in accordance with the GC-MS results where HMR was the main component of the acetone extract, and the minor components can contribute to the spectrum by slightly changing the intensity ratios of the main functional groups. The tissue culture lignin and the milled wood lignin (MWL) showed very similar spectra, the only difference being that the MWL had deeper valleys between the well-resolved peaks (Figure 1). The lignin spectrum resembled quite closely the spectrum of HMR and the acetone extract (Figure 1). However, HMR and the acetone extract exhibited slightly narrower resonance peaks and a sharper offset at 288 eV as compared to the lignins. This spectral feature at 288 eV can be related to C=O C1s→π* transitions [30], since HMR has a lactone moiety in its molecular structure. Dehydroabietic acid was chosen as a representative resin acid in the knotwood cells (Table 3). The C 1s XAS of dehydroabietic acid also exhibited a strong resonance at around 285 eV (Figure 1) due to the benzene ring in its structure. In contrast to lignin and HMR, it did not have a clear peak at higher energies, but rather a sharp edge at 286.7 eV, after which the absorption monotonously rose to ~291 eV. As dehydroabietic acid does not have an aromatic ring with an O substitution, most likely the edge originated from C-C C1s→σ* transitions [31] followed by the C=O C1s→π* transition in the COOH functional group [32]. A tracheid with a deposit with a dehydroabietic acid-type spectrum is presented in the supplementary material (Figure S2).

### 2.4. STXM Imaging of Cryosectioned Samples

**Figure 2:**
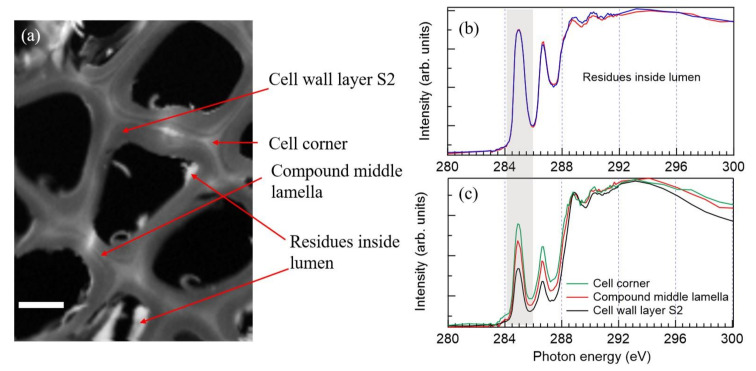
(**a**) An average scanning transmission X-ray microscopy (STXM) image of the cryosectioned knotwood section recorded at the lignin and lignan fingerprint regions at 284.7–285.5 eV. The scale bar is 4 μm. Arrows indicate the areas from where the C 1s XAS presented in (**b**,**c**) were obtained. (**b**) C 1s XAS of two regions with deposits inside the cell lumen. (**c**) C 1s XAS of selected regions: cell corner (green), compound middle lamella (red), secondary cell wall layer S2 (black). The spectra were normalised to have the same intensity at 288.9 eV to better visualise how the increasing lignin concentration is directly visible from the spectrum. The aryl-C,H peak used in the composition analysis has been highlighted with a grey background.

As presented above, the knotwood contains approximately 16% lignans. However, it was difficult to section the knotwood so that the extractives would be preserved inside the cells. During cryosectioning, the knife ripped off some soft material, possibly extractives. In order to highlight the regions which were rich in aromatic compounds (e.g., lignins and lignans), an STXM image was formed by summing several images between 284.7 and 285.5 eV (Figure 2a). The image is presented in optical density, meaning that the white regions absorb strongly and have significant aromatic content, while the black regions inside the cells are empty. As shown in Figure 2b, the lignans were located as residues inside the cell lumina based on their XAS which was remarkably similar to that of HMR. Most probably, these were remains of larger lignan deposits of which majority got removed during sample preparation. The spectra in Figure 2b were obtained by selecting region-of-interests (ROIs) from the stack images by using an aXis2000 program [33], and the approximate points around which the ROIs were selected are shown with arrows in Figure 2a. Even if the C 1s XAS could not separate lignins and lignans with high certainty, the sharper rise of the broad feature starting just below 288 eV agreed better with the lignan spectrum than that of the lignin. A previous study performed with Raman spectroscopy found a high lignan content inside the cell lumen [11], and it is likely that deposits of lignans were observed here.

In C 1s XAS of the wood tissue, the intensity of the double peak structure at 285 and 286.7 eV decreased from the cell corner to the secondary cell wall (Figure 2c). This is in line with the known lignin concentration in these parts of the cell wall [34,35,36,37]. Thus, a semi-quantitative estimation of the lignin concentration can be achieved from XAS. The proportion of lignin could be estimated by assuming that the first aryl-C,H π* peak in those regions originated solely from lignin. The spectrum of the tissue culture lignin was fitted in the energy range 280–300 eV by using symmetric Voigt shape functions for the first sharp peaks and broader features above the ionisation thresholds, which in turn were modelled with inverse tangent functions. The XAS were difficult to fit accurately due to several overlapping ionisation thresholds and resonances, some of which were embedded in the continuum. However, the first π* resonance was quite well separated, and by applying different fitting schemes and by fitting the XAS recorded at two different beamlines, the relative contribution of that peak always remained around 5–6% of the tissue culture lignin used as a reference. A similar fitting method was used before, for example by Boyce et al. [19], who quantified the average chemical structures in the cell walls of recent and fossil plants based on the C edge XAS. In the present study, several ROI XAS were analysed by taking the relative area of the aryl-C,H π* peak with respect to the total area of the fitted spectrum. This ratio was compared to the respective ratio determined from the tissue culture lignin XAS, and the following aromatic compound contents were derived: secondary cell wall S2 layer 38–42%, compound middle lamella 51–57%, cell corner 60–67%. These values were higher than the lignin concentration reported for the Norway spruce stem wood (28.9 ± 0.8% for the total wood cell wall [28]), but especially the non-aromatic content of S2 (58–62%) was well in agreement with the overall carbohydrate concentration in the extract-free knotwood (Table 1). Similar aromatic-content analysis was also made to the lumen-located residues. The analysis based on aryl-C,H π* peak areas showed the content of aromatic substances in these deposits as 79–88%, a value lower than that in pure HMR. This was expected since the extractives of Norway spruce consist of several compounds (Table 3) and the measurement of the cryosection represents this mixture. Cryosectioned samples undergo minimal chemical treatment, and they preserve the chemical information very well. Thus, depending on the type of chemical bonding of the lignans to cell structures and other compounds, their absorption signal could be modified, especially if the bonding is strong. The analysis did not reveal any significant modification of the spectral fingerprints in terms of their energy position or relative intensities, and consequently, the deposits were interpreted to be lignans without significant contribution from other compounds. Here, the accuracy of the XAS-based method is limited by the quality of the reference spectra; however, STXM performed for cryosectioned samples provides a promising technique for the chemical analysis of wood structures even at the sub-100 nm scale. STXM can easily provide qualitative and relative chemical information (e.g., relative amount of aromatic vs. non-aromatic compounds), but the absolute quantification would require a careful calibration of the absorbance using single-component reference samples of a known thickness. This was beyond the scope of the present study since the total amounts of structural compounds and extractives were obtained using other analytical methods.

**Figure 3:**
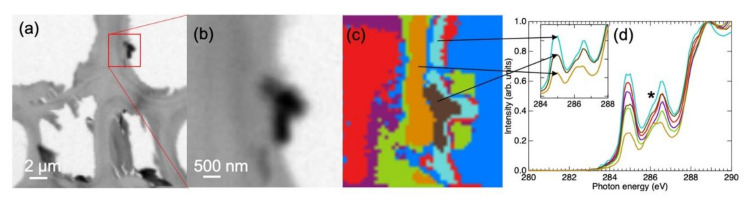
(**a**) An overview image of a cryosectioned Norway spruce section recorded at 320 eV, (**b**) a zoom to a material deposit from where the energy stack was collected, (**c**) a cluster analysis of the material deposit based on the energy region 280–290 eV, (**d**) XAS of clusters with the same colour coding. The spectrum of the empty region depicted in blue in (**c**) is not shown. The inset shows a comparison of three cluster XAS with varying aromatic contribution (highest shown in turquoise). The shoulder of an aryl-O peak has been marked with an asterisk (*).

Another example of the same cryosectioned knotwood sample was selected for a more detailed STXM image analysis with a smaller step size (Figure 3). A principal component analysis performed using MANTIS software found seven principal components (Figure 3c). One of the clusters was an empty area inside the lumen (blue colour in Figure 3c); however, the analysis assigned a weak spectrum for it. The small material deposit inside the lumen seemed to consist of several different areas with their own spectral signatures. Also, the cell wall was divided to several clusters, the major differences being the relative weight of the double π* region compared to the broad continuum region (aromatic vs. non-aromatic components as discussed above). Starting from the leftmost red region (Figure 3c) and ending up to the orange-coloured layer in the middle of the image, the aromatic contribution gradually decreased. This was consistent with the earlier observations of the cell wall, as the lignin concentration is highest in the compound middle lamella [34,35,36,37]. However, the aromatic contribution increased again from the orange-coloured region to the right: the turquoise and red regions in the luminal side of the cell wall had the highest aromatic content and are possibly associated with a thin extractive layer as discussed later. The brown region also had a significant aromatic contribution. As it would be surprising to find lignin in significant amounts in this region, it is possible that these areas contain deposits of lignan not swiped away during cryosectioning.

The XAS exhibited a shoulder in the aryl-O C 1s→π* peak towards lower energies (Figure 3d). This was not observed in the reference spectra shown in Figure 1 and not as intense in the measurement of Figure 2. The shoulder was especially clear in the thin (~0.5 μm) layer marked with orange colour in Figure 3c, whose spectrum (Figure 3d, inset; orange solid line) exhibited a very broad second peak starting from 286 eV. The cell wall spectrum of an aspen (*Populus*) recorded by Jereminc et al. [21] shows a similar asymmetric second peak. Many functional groups have been reported to have C 1s→π* transitions in the energy region 286–287 eV, for example, phenols and quinones [32] and aldehydes and ketones [38]. There are two possible explanations for this broadening: it can be either due to the different chemical compositions in different parts of the cell wall, or that X-ray exposure alters the chemical structure during the measurement. The effects of the X-ray beam spot size and the dwell time on tissue sections of oak and on cellulose acetate have been systematically investigated [17]. In the oak sections, lignin was observed to be more abundant in the compound middle lamella than in the rest of the cell wall. Furthermore, a clear redshift in the aryl-O C 1s→π* peak was observed in the secondary cell wall S2 layer as compared to the compound middle lamella [17]. Also, there was an increase in the relative intensity of the aryl-O peak, which is in accordance with the information that lignin in the secondary cell wall is formed from monomers with a higher O-substitution degree of the aromatic ring as compared to lignin in the compound middle lamella [34,35,39]. However, similar spectral changes were seen in the cellulose acetate samples as a function of the X-ray dose: a high dose resulted in a redshift and a relative intensity increase of the aryl-O C 1s→π* peak [17]. It was suggested that photoinduced chemical reactions modify the structure of polysaccharides more likely than those of more stable aromatic compounds such as lignin. This is in line with our observation that the spectral weight of the redshifted aryl-O peak was connected to the increasing amounts of polysaccharides in the studied region. The photoinduced alteration is also a plausible explanation, because the spectra of the image stack in Figure 2, recorded with a larger step size and a smaller dwell time, did not show similar signatures of the radiation damage. Furthermore, the energies above 290 eV in the energy stack of Figure 3 showed an indication that the studied structure changed its morphology during the X-ray exposure. Thus, the cluster analysis only concentrated on the energies 280–290 eV, where, by visual means, the sample seemed to stay intact. The molecular level radiation damage can undoubtedly start to occur before the changes in morphology are visible.

### 2.5. STXM Imaging of Resin-Embedded Samples

The STXM imaging of cryosectioned samples showed lignan-type residues inside the cell lumina. However, the sample preparation was not able to conserve the cell structures intact, and it remained unclear whether lignans form specific structures in the lumina. Glutaraldehyde-paraformaldehyde fixation and resin-embedding are known to preserve the structural features of cells and were also applied here expecting that they would also fix lignans to their original location in the tissue. The samples were stained with OsO_4_ so that the same sections could be used in TEM analysis as well. Resin-embedded samples contained lignans as a continuous distribution in some cell lumina and in specific deposits together with Os. As shown in [12], highly absorbing, well-defined material deposits with some aromatic compounds were observed; these are now believed to be modified lignans which had reacted during the sample preparation with OsO_4_. In order to reveal the uniform distribution of lignans in one tracheid lumen, data analysis with the careful subtraction of the resin background was needed. The definite differentiation of aromatic compounds lignin and lignan was possible using two complementary STXM measurements, one performed at the C 1s edge and one at the O 1s edge.

**Figure 4:**
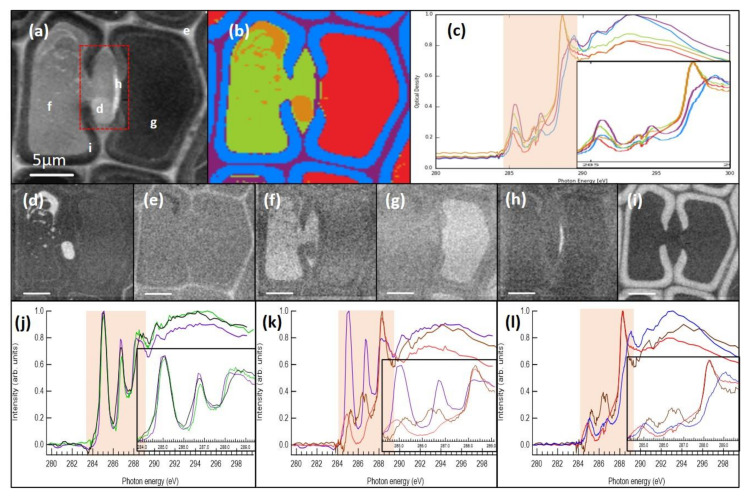
Cell structures of the resin-embedded knotwood tracheids with some deposit material measured at the C 1s absorption edge. (**a**) Average optical density map of STXM measurement with measurement locations for the structural spectra (**d**–**i**); the bordered pit pair is marked with a red square, (**b**) cluster analysis of the STXM measurement, (**c**) average cluster spectra of the cluster analysis. Localisations of (**d**) the measured deposit in the bordered pit chamber, (**e**) cell corner and compound middle lamella, (**f**) left tracheid, (**g**) right tracheid, (**h**) torus, (**i**) secondary cell wall spectra. (**j**) Spectra of the epoxy-resin-subtracted left tracheid (green), the reference lignan HMR (purple) and the tissue culture lignin (black). (**k**) Spectra of the epoxy-resin-subtracted deposit material (brown), the reference epoxy resin (red) and HMR (purple). (**l**) Spectra of the epoxy-resin-subtracted deposit material (brown), the epoxy resin from the tracheid **g** (red) and the secondary cell wall (blue). The scale bars in all images are 5 µm.

**Figure 5:**
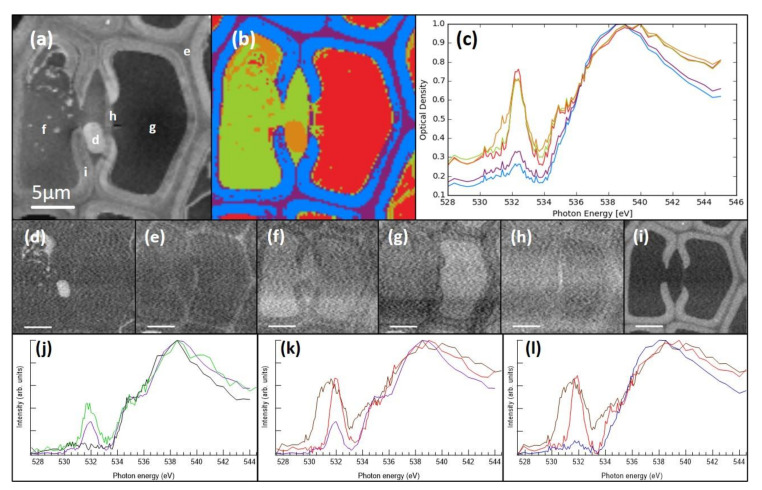
Cell structures of the resin-embedded knotwood tracheids with some deposit material measured on O 1s. (**a**) Average optical density map of STXM measurement with measurement locations for the structural spectra (**d**–**i**), (**b**) cluster analysis of the STXM measurement, (**c**) average cluster spectra of the cluster analysis. Localisations of (**d**) the measured deposit in the bordered pit chamber, (**e**) cell corner and compound middle lamella, (**f**) left tracheid, (**g**) right tracheid, (**h**) torus and (**i**) secondary cell wall spectra. (**j**) Spectra of the epoxy-resin-subtracted left tracheid (green), the reference lignan HMR (purple) and the tissue culture lignin (black). (**k**) Spectra of the epoxy-resin-subtracted deposit material (brown), the reference epoxy resin (red) and the HMR (purple). (**l**) Spectra of the epoxy-resin-subtracted deposit material (brown), the epoxy resin from the tracheid **g** (red) and the secondary cell wall (blue). Scale bars in all images are 5 µm.

Even though the epoxy-resin-embedding seemed to preserve the structures better, it complicated the spectral analysis of the deposits, since the spectral features were superimposed on top of the spectrum of the embedding resin. Similar to an earlier observation [17], the resin used in the present study did not penetrate the cell walls, and thus, did not create a constant background. Hence, the subtraction of the resin signal was a challenging task. Examples of the resin-embedded sample recorded at C 1s and O 1s edges are presented in Figure 4 and Figure 5, respectively. In tracheids, the cell lumina were usually empty and thus filled with the embedding material. The average optical density maps at C 1s and O 1s show a clear difference in absorption between the left and the right cell lumina (marked with f and g in Figure 4a and Figure 5a), indicating that the left cell and the bordered pit chamber were not empty when the sample was prepared. This is further highlighted in Figure 4b and Figure 5b, which present the cluster analysis, and Figure 4c and Figure 5c, which present the average cluster spectra at the C 1s and O 1s edges, respectively.

The right-side tracheid in Figure 4 had a spectrum that matches the spectrum of the epoxy resin, whereas the left-side tracheid had an additional peak at 286.8 eV which matches the typical energy of the second peak in lignans and lignins. To resolve the composition of the material embedded in the epoxy resin, the spectrum of the resin was subtracted from the spectrum of the ROI. For this, the relative proportion of the epoxy resin is important. In the case of the cell wall, there was no need for this subtraction as the resin did not penetrate there. The basis of determining the amount of epoxy resin signal to be subtracted was done by minimising the typical peak in the spectrum at 288.3 eV while making sure that there were no negative values produced. In the case of the left tracheid in Figure 4, the relative amount of the spectrum of the epoxy resin removed was 90%. The spectrum left after this subtraction was similar to that of the reference HMR and the tissue culture lignin, the energy region at 288 eV following closer to the HMR spectrum (Figure 4j). O 1s measurements were used to further confirm that the material is lignan. Similar to that in the material deposits, lignan has a clear peak at 532.0 eV, whereas in lignin, the peak is almost nonexistent (Figure 5j). This indicates that the left tracheid had some residual lignan in the cell lumen. The unmodified spectrum of the deposit in the bordered pit chamber had similarities to that of the epoxy resin which is most likely due to the epoxy resin impregnating the deposit material. This observation means that the properties of the deposited material differ from those of the cell wall where epoxy resin does not penetrate. A strong peak in the deposit material was observed at 288.3 eV even after completely subtracting the measured epoxy resin spectrum (Figure 4k). This means that the deposit had carbonyl/carboxyl groups in its structure, and hence, it is likely that the deposit contains other compounds in addition to the lignans detected. The locations of the first and the second peak did not match those observed for the lignan deposits in the cryosectioned samples; there was a clear shift in peak energies (Figure 4k). O 1s XAS of the deposit had a peak at 532.5 eV (Figure 5k) indicating that it was not lignin. The peak was wider than that in the reference lignan and it also had a shift of 0.4 eV to lower energy. The differences in both C 1s and O 1s XAS compared to lignan can be due to chemical changes caused by fixation, embedding and/or staining of the samples. There was also a problem with the top-up procedure during the C 1s edge measurement. The data had to be compiled from two sets which caused a mismatch in the absorption values in the spectra around 286.5 eV (Figure 4l). This made the localisation of the maximum of the second peak uncertain. At the O 1s edge, the lignin-rich layers within the cell wall did not show a strong peak at 532.0 eV. This indicates that lignan and lignin can be differentiated. The O 1s had a poorer signal-to-noise ratio compared to C 1s which can be seen in Figure 4 and Figure 5, as there was significantly less O than C in the samples.

**Figure 6:**
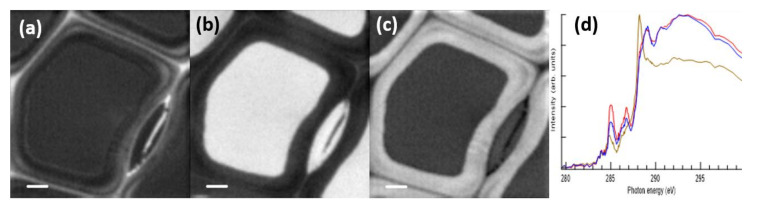
The extracted C 1s XAS and their localisation in a resin-embedded knotwood cell. (**a**) Localisation of the cell corner- and the compound middle lamella-type of spectra. (**b**) Localisation of the epoxy resin spectrum. (**c**) Localisation of the secondary cell wall spectrum. (**d**) Spectra of different ROIs: cell corner and compound middle lamella (red line), epoxy resin extracted from the middle of the cell lumen (brown line), secondary cell wall (blue spectrum). Scale bar is 2 μm.

Unexpectedly, C 1s XAS of the torus of the bordered pit pair showed a clear signal of the aromatic compounds (Figure 4), and the cluster analysis assigned it to the same cluster as the cell corners and the compound middle lamella. Another bordered pit pair was also measured (Figure 6), confirming the findings in Figure 4. Again, the torus exhibited a clear double peak structure in the aromatic fingerprint region (Figure 6d, red spectrum). In Norway spruce sapwood, TOF-SIMS observations have detected negligible lignin contribution in the tori [40]. Raman images derived by using vertex component analysis of the sapwood of Norway spruce, on the other hand, showed that the secondary cell wall S3 layer and the tori have similar molecular compositions with changes observed in the lignin composition as compared to other parts of the cell wall [37]. Lignin in the tori of coniferous trees was also detected by Sachs (1963), who discussed that this is due to the maturation of the pit membranes [41]. This is likely the case in the knot heartwood of Norway spruce, where water transport is not the function anymore and the tracheids have been filled with extractives to be used for protection. Thus, the current findings also give a new insight to the torus composition.

### 2.6. STEM-EDS Imaging of Resin-Embedded Samples

As shown above, in some cases, the resin-embedded samples showed a rather uniform distribution of lignan in the cell lumina, but also had clear, highly absorbing deposits. The spectral fingerprints of these deposits had some similarities with the reference lignan HMR, but the double peak fingerprint structure had a different energy splitting. The very high contrast in the STXM images below the C 1s absorption edge indicated that the deposits contained heavy elements, for example, Os from the OsO_4_ staining. It was assumed that OsO_4_ reacted with lignans, and this chemical reaction reduced Os while the lignan was oxidised, thus destroying its C 1s XAS spectral fingerprints due to a decrease in the double bonds. OsO_4_ has been observed to react strongly with low molecular weight phenolic compounds which are similar to lignin precursor molecules, but to a lesser extent with MWL, and only very slightly with wood sections of radiata pine (*Pinus radiata*) [42]. An experiment was conducted in a test tube by mixing OsO_4_ solution with dry HMR powder with a light yellowish colour, and a blackish-brown sediment was formed (Figure 9f). The reaction between HMR and OsO_4_ was rapid and visually observed. When the tissue culture lignin was tested with OsO_4_, no reaction was seen, but on the other hand, the tissue culture lignin had a blackish-brown colour before OsO_4_ was added.

**Figure 7:**
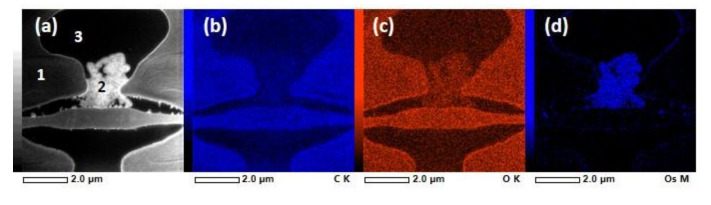
STEM-EDS of resin-embedded knotwood tracheid with some deposit material. (**a**) STEM image with region numbers corresponding to Table 4. EDS images showing (**b**) C K α, (**c**) O K α and (**d**) Os M α X-ray emission.

**Figure 8:**
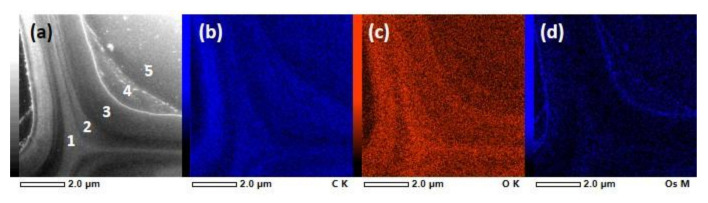
STEM-EDS of the resin-embedded knotwood tracheid’s cell wall structure. (**a**) STEM image with region numbers corresponding to Table 5. EDS images showing (**b**) C K α, (**c**) O K α and (**d**) Os M α X-ray emission.

STEM-EDS imaging performed on the same cells (but different sections) further confirmed the accumulation of Os to the regions inside cell lumina that were strongly absorbing in the STXM experiments. We stress that in contrast to STXM, the intrinsic line-broadening of the fluorescence signals used in the STEM-EDS analysis prohibits any chemical information to be retrieved (i.e., both aromatic and non-aromatic carbon compounds contribute to the same total carbon signal), and consequently, STEM-EDS provides complementary information about the quantitative elemental composition. Figure 7 and Figure 8 present STEM-EDS images together with elemental analyses of different regions in Table 4 and Table 5, respectively. Figure 7 shows a STEM-EDS analysis of a bordered pit region close to the region imaged using STXM in Figure 4 and Figure 5. The secondary cell wall (region 1) had 93 atomic-% of C and 7 atomic-% of O, and almost no Os. The secondary cell wall and the torus seemed to have rather uniform C and O distribution (Figure 7a,b). As can be seen from Figure 7d, Os was concentrated on the deposit (region 2), and the EDS analysis revealed a significant increase in Os concentration as compared to the cell wall (region 1) or the lumen (region 3). The cell lumen (region 3) had a different C-to-O ratio as compared to the secondary cell wall, which is natural, since the lumen was mostly filled with the embedding resin. STEM-EDS analysis of the cell corner region (Figure 8 and Table 5) was conducted to further confirm that the Os did not react with lignin. The amount of Os stayed very low in all the regions, and the relative amounts of C and O showed a slight increase in C when moving away from the cell corner towards the secondary cell wall. In Figure 8, the regions 4 and 5 had similar atomic composition as the cell lumen and mostly contained the embedding resin. A close look at Figure 7d and Figure 8d revealed that Os was also present in a thin layer on the luminal surface of the cell wall. A similar electron dense layer, called the extractive layer, was detected in the TEM study of KMnO_4_-stained sapwood sections of the Norway spruce [43]. The wart contents have been considered as remnants of lignin precursors brought to the inner wall surface at the end of tracheid differentiation [44]. These remnants are possibly deposited and polymerised in the warts. The cluster analysis of the STXM experiments (Figure 3c, Figure 4c and Figure 5c) assigned a thin layer on the luminal surface of the cell wall. Even if the cluster analysis of such a thin layer was challenging, it was consistently assigned with spectra containing aromatic groups, supporting the idea of an extractive layer.

## 3. Materials and Methods

Two Norway spruce trees were cut in southern Finland (60°21′22.8″N 25°01′46.7″E) on 21 October 21 2016, and 23 January 2018, as per resin-embedding and cryosectioning sample preparation protocols, respectively. For the resin-embedded samples, a whorl was sawn off at a height of 2.6 m from a 54-year-old tree, and, for cryosectioned samples, a whorl was sawn off at a height of 3.42 m from a 44-year-old tree. Both sample whorls were sawn from the second whorl above the crown height, where lignan content is high [3]. Immediately after the trees were harvested, the knots were roughly prepared from the whorls, put on solid carbon dioxide ice (−78.5 °C), transported to the laboratory and stored at −80 °C.

**Figure 9:**
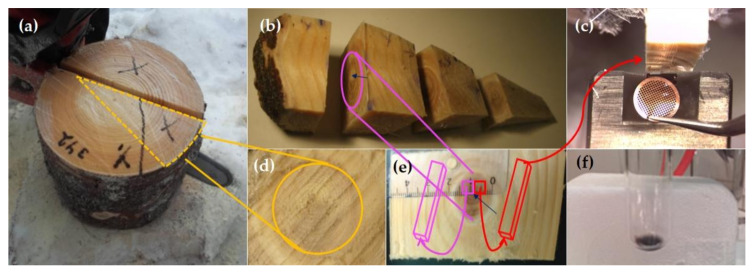
Tree sampling, knotwood preparation and a chemical reaction between HMR and OsO4. (**a**) A stem wood disc with knotwood inside the stem. A sector including knotwood was sawn (marked in yellow). (**b**) A 3 cm thick knotwood disc was taken 2 cm from the bark. Blue arrow shows the knotwood pith. (**c**) An ultramicrotome was used to cut the knotwood transverse surface. (**d**) Transverse cutting surface of a branch base. (**e**) Knotwood sticks (pink and red) were cut above and close to the knot pith (marked with a blue arrow). (**f**) Reaction of HMR with OsO4.

In the laboratory, the knots were kept cool while prepared (Figure 9) and cut into four equal-size discs. The third disc from the pith was prepared for GC-MS analysis to determine extractives, and for STXM analysis and STEM-EDS imaging. Then, two 5 × 5 × 20 mm sticks were taken side by side in a longitudinal direction from the upper knot part, which did not contain any of the compression wood that is typical for the lower part of the knotwood.

### 3.1. Chemical Analyses

#### 3.1.1. GC-FID/GC-MS Analysis of Extractives

The knotwood sample sticks that remained after preparing the cryosectioned samples were used for gas chromatographic analysis with a flame ionisation detector (GC-FID) and compounds were identified with GC mass spectrometry (GC-MS). The purpose for this was to use as identical knotwood material as possible for both analyses (Figure 9e). The knotwood sticks were dried for 12 h at −20 °C in a vacuum desiccator, stored at −80 °C and then milled into fine powder with a Polymix mill at −20 °C. The knotwood powder was sequentially extracted with a mini-Soxhlet apparatus using hexane (6 h, ca 70 percolations, 5 min per percolation) and acetone (6 h, ca 70 percolations, 5 min per percolation). The extraction of knotwood samples with acetone only (6 h, 70 percolations, 5 min per percolation) was performed as well.

The extracts were evaporated in a stream of N2 at 40 °C, dried for 30 min at 40 °C in a vacuum desiccator and weighed. The dried extractives were stored at −20 °C in the dark until further analysis.

Ten mg of dried acetone extractives were re-dissolved in 10 mL of acetone (p.a. grade) to obtain the corresponding stock solution. Exactly 0.5 mL of the extract was transferred into a 10 mL test tube equipped with a hermetically sealing Teflon-coated screw cap. Two mL of internal standards in methyl-tert-butyl ether (MTBE) solution, containing exactly 0.02 mg/mL of heneicosanoic acid, cholosteryl heptadecanoate, 1,3-dipalmitoyl-2-oleoylglycerol (Sigma Chemical Co., St. Louis, MO, USA) and betulinol (isolated and purified in the Laboratory of Wood and Paper Chemistry at Åbo Akademi University, Turku, Finland) was added, and the tube content was evaporated with a N2 flow on a water bath at 40 °C. After additional drying in a vacuum desiccator at 40 °C for 30 min, the extractives were silylated with 160 µL of BSFTA:TMCS:pyridine (4:1:1 *v*/*v*/*v*) at room temperature overnight in the dark.

The extractives were analysed by a GC method using short and long capillary columns. The group analysis of high-boiling sesqui-/dilignans, steryl esters and triglycerides was performed with the short column. The long column was used to determine the component composition of lignans, fatty and resin acids, as well as the other relatively low-molar-mass compounds. The GC-MS analysis was used to confirm the component identification to support the long column GC analysis.

The GC analysis on the short column was performed with a GC instrument Perkin Elmer Clarus 500 equipped with a capillary column HP-1 (7 m × 0.53 mm, film thickness 0.15 µm). The protocol for the column oven was as follows: starting temperature 100 °C, hold time 0.5 min, temperature increase rate 12 °C/min, end-temperature 340 °C, hold time 5 min. The injector was a programmable evaporator with the protocol: starting temperature 80 °C, hold time 0.1 min, temperature increase rate 50 °C/min to 110 °C, then the rate of 15 °C/min, end-temperature 330 °C, hold time 7 min. Hydrogen with the flow rate of 7 mL/min was used as a carrier gas. The GC instrument equipped with an FID was heated at 350 °C. The sample volume was 3 µL (direct injection into the column).

The GC analysis on the long column was performed with a GC instrument Perkin Elmer Auto SystemXL equipped with capillary columns: channel A-HP-1 (25 m × 0.2 mm, film thickness 0.11 µm); channel B-HP-5 (25 m × 0.2 mm, film thickness 0.11 µm). The protocol for the column oven was as follows: starting temperature 120 °C, hold time 1 min, temperature increase rate 6 °C/min, end-temperature 320 °C, hold time 15 min. The injector was a programmable evaporator with the protocol: starting temperature 160 °C, temperature increase rate 8 °C/min, end-temperature 260 °C, hold time 15 min. Hydrogen with the flow rate of 0.8 mL/min (20 mL/min including split) was used as a carrier gas. The GC instrument was equipped with an FID heated at 310 °C. The sample volume was 3 µL (split 1:24).

In order to confirm the component identification with the long column, the GC-MS analysis of the extractives on a HP 6890-5973 GC-MSD instrument was applied. The GC-MS analysis was performed with the HP-1 capillary column, at conditions similar to those used for the GC-FID instrument. The compounds were identified as silylated derivatives, by comparing the mass spectra of their chromatographic peaks with the spectra of pure compounds from the in-house Spectral Library and the commercial Wiley 10th/NIST 2012 spectral library.

#### 3.1.2. Hemicelluloses and Pectins

The hemicellulose and pectin content were determined according to [45]. Shortly thereafter, 8–12 mg of pre-extracted (both hexane and acetone/water) knotwood powder was placed in a pressure-resistant, pear-shaped flask and 2 mL 2 M solution of HCl in anhydrous MeOH was added. The sample was kept at 105 °C for 5 h. One mL of a calibration solution containing 0.1 mg/mL of arabinose (Ara), glucose (Glc), glucuronic acid (GlcA), galactose (Gal), galacturonic acid (GalA), 4-O-methyl glucuronic acid (4-O-Me-GlcA), mannose (Man), rhamnose (Rha) and xylose (Xyl) in methanol was evaporated to dryness and treated for 3 h in the same method as outlined above. Subsequent to cooling down to room temperature, 80 µL of pyridine was added to neutralise the solution, and the flask was shaken thoroughly. Four mL of internal standard containing 0.1 mg/mL resorcinol in methanol was added and the sample was shaken. An aliquot of 1.0 mL of the clear solution was evaporated to dryness under a N2 stream. The dried sample was silylated using a solution containing 120 µL pyridine, 150 µL hexamethyl disilazane (HMDS) and 70 μL trimethylsilyl chloride (TMCS). The silylation was carried out overnight at room temperature. The silylated samples were analysed by GC-FID (Shimadzu GC-2010, Kyoto, Japan) with HP-1 Column (25 m × 0.2 mm I.d., film thickness 0.11 µm). The temperature profile was 100 °C -> 175 °C, 4 °C/min, 175 °C -> 290 °C, 12 °C/min. The injector temperature was 260 °C and the detector temperature 290 °C. The following correction factors were used when calculating the results: Man, Glc and Gal 0.9, Ara and Xyl 0.88, Rha 0.89, GlcA, GalA and 4-O-Me-GlcA 0.91. All the analyses were carried out using two replicates.

#### 3.1.3. Cellulose

The cellulose content was determined by a two-stage acid hydrolysis followed by GC-FID. Pre-extracted knotwood powder (10 mg) was transferred into a test tube with a glass ball, 0.2 mL of 72% sulfuric acid was added, and the sample placed in a vacuum oven at 40 °C. The pressure was dropped to 0 bar and increased back to normal pressure after a few seconds. The sample was placed on a fume hood and left to stand for 2 h at room temperature. Deionised water (0.5 mL) was added and incubation continued for 4 h. The first hydrolysis was finalised by adding 6 mL of deionised water, and the sample was left to stand in the fume hood overnight at room temperature.

The secondary hydrolysis was carried by autoclaving the sample at 120 °C for 90 min. After the hydrolysis, the sample was cooled down to room temperature. Two drops of bromocresol green were added as an indicator, and the sample was neutralised using BaCO_3_. The colour changed from yellow to blue when neutralisation had occurred. One mL of sorbitol (5 mg/mL) in deionised water was added as an internal standard. The tube was centrifuged, and an aliquot of the clear solution was transferred to a new tube and evaporated to dryness under a N2 stream. The dried sample was silylated similarly to the samples in acid methanolysis. Cellulose powder made from cotton linters (Sigma-Aldrich Co., St. Louis, MO, USA) was used for calibration, and treated in the same way as the knotwood sample. The cellulose content was determined with the same GC-FID system as glucose in acid methanolysis (see above). The cellulose content of the sample was obtained by subtracting the amount of (anhydro) glucose obtained by acid methanolysis-GC from the amount of glucose obtained by acid hydrolysis. All the analyses were carried out using two replicates.

#### 3.1.4. Lignin

The klason lignin in pre-extracted and dried knotwood was determined by acid hydrolysis using 72% sulfuric acid according to a modified Klason lignin method described by Schwanninger and Hinterstoisser [46]. Acid-soluble lignin was determined by UV absorption method in accordance with TAPPI UM250 at 205 nm.

### 3.2. Resin-Embedded Samples

Two 1 mm3 samples close to the knot pith were prepared from the knotwood and fixed for 4.5 h in 2.5% glutaraldehyde and 2.2% paraformaldehyde in 0.1 M Na-phosphate buffer, pH 7.4. After a three-day storage in 0.1 M Na-phosphate buffer, one of the samples was stained with 2% OsO_4_. Both samples were washed in ascending ethanol and acetone series and embedded in LV Resin (TAAB LOW VISCOSITY PREMIX KIT, TAAB Laboratories Equipment Ltd., Aldermaston, UK). Samples were cut into 60–120 nm thick transverse sections with an ultramicrotome and placed on TEM copper slots with a Pioloform foil window.

### 3.3. Cryosectioned Samples

A knotwood stick (5 × 5 × 20 mm) containing annual rings 1–5 from the knot pith was frozen in liquid nitrogen. First, the transverse surface was trimmed at −70 °C with a diamond knife using Leica EM UC7 ultramicrotome (Leica Microsystems, Wetzlar, Germany) to shape a raised area (1 mm × 1 mm) in the 4th annual ring from the knot pith. Then, sections (100 and 140 nm) were cut from the transverse, pre-trimmed surface with a diamond knife (Diatome Cryo 35°) using the ultramicrotome at −70 °C, and collected with the aid of an eye lash coated with gold on 175 mesh copper grids (G175-CU, Electron Microscopy Science, Hatfield, PA) without any supporting film, and stored at −80 °C. The sections were attached to the grid using Leica EM Crion, while the tweezers holding the grid were attached to the microtome body having zero potential [16,47].

### 3.4. Reference Samples

The reference samples were diluted in distilled water or ethanol, and a drop casted on silicon nitride windows (NX5100C, Norcada Inc., Edmonton, AB, Canada) for X-ray microscopy.

The following reference samples were prepared, or commercial references were used:

1. Hydroxymatairesinol: a mixture of both isomers HMR2 and HMR1 (93:7, *w*/*w*), approximately 90% purity (obtained from Åbo Akademi University, Turku, Finland). The isolation was carried out according to Eklund and Raitanen (2019) [48].
2. Tissue culture lignin: extracellular lignin was collected from a Norway spruce cell suspension culture (line A3/85) [49,50,51]. Lignin was pelleted by centrifugation from the culture medium and washed with water. Lignin-bound proteins [52] were extracted with buffered 1 M NaCl, after which the carbohydrates bound to lignin [53] were diminished by a treatment with glycosyl hydrolases according to Warinowski et al. (2016) [52]. After several washes with water, the lignin was lyophilised.
3. Milled wood lignin (MWL) was prepared from Norway spruce sapwood according to the Björkman procedure (1956) [54].
4. Microcrystalline cellulose, CAS Number: 9004-34-6 (Sigma-Aldrich Co., St. Louis, MO, USA).
5. Norway spruce galactoglucomannan (GGM) with residual arabinoglucurunoxylan and pectin was extracted with pressurised hot water, concentrated and precipitated with ethanol [55].
6. Dehydroabietic acid, CAS Number: 1740-19-8 (Sigma-Aldrich Co., St. Louis, MO, USA).
7. Acetone extract of the Norway spruce knotwood: the knotwood extract was prepared according to the protocol presented in Section 3.1.1.

### 3.5. Scanning Transmission X-ray Microscopy

Cryosectioned samples were imaged at the HERMES beamline at the Synchrotron SOLEIL (Saint-Aubin, Gif-sur-Yvette, France) [56]. The image stacks were measured with varying energy steps. At C 1s edge, the measurement energy range 280–282.8 eV was measured with a step of 1.4 eV, 283 Ev–292 eV with a step of 0.1 eV, and again above 292 eV with an increasingly coarse step from 1 to 2 eV. In the stack presented in Figure 2, the spatial step size was 200 nm and the dwell time 3 ms, and in Figure 3, the step size of 50 nm and the dwell time of 5 ms were used. For experiments performed at the O 1s edge, the region 514.3–524.5 eV was measured with a step of 0.5 eV, the region 524.6−545.3 eV with a step of 0.1 eV, and 545.8–554.3 eV with a step of 0.5 eV. Dwell time was 5 ms.

Resin-embedded samples were studied at the BL4U beamline at the UVSOR III storage ring (Okazaki, Japan) [57]. The stacks were measured with varying energy steps. For the C 1s edge, in the region 280–284 eV a step of 0.25 eV was used, in 284.1–290 eV a step of 0.05 eV was used, in 290.2–293 eV a step of 0.2 eV was used and in 293.5–300 eV a step of 0.5 eV was used. For O 1s the region 528–530 eV was measured with a step of 0.4 eV, 530.1–537 eV with a step of 0.1, eV and 537.5–545 eV with a step of 0.5 eV. The dwell time was 3 ms, and the spatial step sizes were 200 nm (Figure 6) and 250 nm (Figure 4 and Figure 5). Absolute energy calibration was not performed, and two different data sets obtained at the SOLEIL and the UVSOR facilities were calibrated by recording the same reference spectra and setting the aryl-C,H peak to 285.0 eV, agreeing well (within ~0.2 eV) with the transitions of respective functional groups reported in the literature. The O 1s XAS was calibrated by setting the C=O peak to 532.0 eV as reported in [18].

The cryosectioned knotwood xylem samples contained several cell types in the same section, and the results were consistent when similar cells were selected, but clearly differed when a cell of a different type was selected as shown in the supplementary material (Figure S2). The resin-embedded samples recorded at C 1s and O 1s edges showed consistent results and similar deposit structures than reported in [12]. Furthermore, the two tori analysed (Figure 4 and Figure 6) showed reproducible results about the torus composition.

### 3.6. Scanning Transmission Electron Microscopy with Energy Dispersive Spectroscopy

STEM-EDS analysis was carried out at the Centre for Material Analysis (University of Oulu, Oulu, Finland), using JEOL JEM-2200FS transmission electron microscope (JEOL Ltd., Tokyo, Japan) with 200.0 kV acceleration voltage and 1.0 nA current. STEM images were taken with a high-angle annular dark field (HAADF) detector enhancing the contrast between the low- and high-Z elements. Elemental analysis was done by detecting fluorescent photons in the energy range of 0–40 kV with a JEOL Dry SD100GV (JEOL Ltd., Tokyo, Japan) detector. A thin (approximately 15 nm) layer of carbon was evaporated on the TEM grids to ensure electrical conductivity.

### 3.7. OsO_4_ Binding Experiment

OsO_4_ binding to HMR was tested in a test tube. Then, 1% aqueous solution of OsO_4_ (CAS No. 20816-12-0, Electron Microscopy Sciences, Hatfield, PA, USA) was added to an HMR powder. The colour of the reaction solution was visually observed immediately, after 1 min and after a 1 h incubation at room temperature (Figure 9f). A similar test was carried out for the lyophilised tissue culture lignin.

### 3.8. Data Analysis

The data analyses of STXM experiments were carried out with aXis2000 software (Hamilton, ON, Canada) [33] and the cluster analysis with the MANTiS multivariate analysis tool (2nd Look Consulting, Hong Kong, China) [58,59]. XAS were fitted using the SPANCF macro package [60,61] written for Igor Pro software (WaveMetrics, Inc., Portland, OR, USA).

## 4. Conclusions

Norway spruce knot heartwood is an ideal research object to study how phenolic extractives and structural compounds can be distinguished in their native environment. In this work, the purpose was to develop methods to localise native lignans in the xylem, to obtain an insight to the molecular structure of the wood and resolve whether wood extractives are connected to cell wall components. We utilised an advanced, synchrotron radiation-based characterisation method, STXM, for its potential to reveal authentic properties of these compounds in situ. Our results show that soft X-ray STXM is a promising tool for even sub-100 nm chemical characterisation of the wood. When HMR was used as a model compound, lignan was recognised and located inside the tracheid lumina as continuous distribution, but also confined to specific deposits inside the lumina and in the bordered pit chambers. The STXM results were supported by STEM-EDS, which confirmed the accumulation of osmium to lignan deposits. We did not observe any indications of lignans being present in the cell wall or having a strong connection to the cell wall structure. Hereafter, the high spatial resolution of STXM could be exploited to study, for example, the gradual changes in lignin subunit concentrations in the cell wall. This requires a systematic study of the radiation damage to avoid X-ray-induced structural and chemical changes. With STXM, it was possible to detect compounds with a high level of similarity in intact wood cells. Sample preparation is a crucial step, since very thin, loosely attached, unfixed and cryosectioned native structures changed their form within the STXM chamber, which was a challenge in the imaging analysis. In the future, lowering the cutting temperature or cutting thicker sections could be tested on samples with both hard and soft structures. The resin-embedding preserved the cell structures better but altered the chemical composition of the low molecular weight aromatic compounds. The sample preservation will benefit from the ongoing development of novel in situ cells allowing the experiments to be carried out in a more native state, unleashing the full potential of chemical imaging by STXM.

## Figures and Tables

**Table 1:** Carbohydrates’ composition of the knotwood as anhydrosugars.

Component	mg g^−1^ Knotwood
Cellulose	369.6
Non-cellulosic sugars	
Mannose	83.7
Glucose	36.4
Galactose	37.1
Xylose	61.6
Arabinose	14.4
Rhamnose	1.8
Glucuronic acid	1.5
Galacturonic acid	11.7
4-*O*-Methylglucuronic acid	6.7
Total non-cellulosic carbohydrates	255.0
Total carbohydrates	624.5

**Table 2:** The amounts of extractives in the acetone and hexane extracts in the knotwood samples. Location of the sample in the crown is indicated.

Sample Knot ID	Extraction Solvent	Knotwood Extractives (mg g^−1^)
Eastern knot ^1^	Acetone only	208.8
Eastern knot ^1^	Hexane	4.0
Northern knot ^2^	Acetone only	181.9
Northern knot ^2^	Hexane	4.5
Eastern knot ^1^	Acetone ^3^	194.43
Northern knot ^2^	Acetone ^3^	174.43

^1^ Additional knot of the same whorl, which was chosen for STXM; ^2^ Knot that was used for STXM (cryosectioned sample); ^3^ Acetone extract after pre-extraction with hexane.

**Table 3:** Compounds present in the knotwood extracts.

Chemical Component	Eastern Knot ^1^	Northern Knot ^2^	Eastern Knot ^1^	Northern Knot ^2^
	Acetone Extract (%)		Acetone Extract after Pre-extraction with Hexane (%)	
Acid C15:0	data	0.00	0.00	0.00
Acid C16:1	0.01	0.00	0.01	0.01
Acid C16:0	0.00	0.08	0.00	0.01
Acid C18:3	0.03	0.03	0.00	0.01
Acid C18:2	0.04	0.08	0.01	0.03
Acid C9-18:1	0.03	0.05	0.02	0.03
Acid C11-18:1	0.01	0.03	0.00	0.00
Dehydroabietic acid	0.07	0.09	0.03	0.02
Acid C20:3	0.04	0.00	0.00	0.00
Acid C22:0	0.03	0.03	0.03	0.06
Acid C23:0	0.03	0.00	0.00	0.03
Acid C24:0	0.08	0.03	0.05	0.04
**Sum of fatty and resin acids**	**0.37**	**0.43**	**0.17**	**0.24**
7R-Todolactol	1.23	1.24	1.04	1.39
Secoisolariciresinol	3.30	4.17	3.81	4.04
7S-Todolactol	3.35	3.26	2.78	3.30
7R-Isoliovil	0.69	0.66	0.63	0.55
α-Conidendric acid	2.13	1.79	2.23	1.69
7`-Hydroxymatairesinol	3.95	4.03	3.84	3.67
Hydroxymatairesinol HMR1	21.72	23.29	23.68	23.24
Hydroxymatairesinol HMR2	37.26	36.42	37.23	36.57
α-Conidendrin	2.23	2.24	2.74	2.07
9´-Hydroxymatairesinol	0.88	1.02	0.58	1.08
7`-Oxo-matairesinol	0.36	0.28	0.40	0.35
Lariciresinol	0.94	0.97	0.78	0.96
iso-Hydroxymatairesinol	2.47	2.38	2.28	2.41
7`-Oxolariciresinol	0.34	0.32	0.31	0.29
epi-iso-Hydroxymatairesinol	0.38	0.38	0.38	0.37
7R-Todolactol	1.23	1.24	1.04	1.39
**Sum of lignans**	**81.23**	**82.45**	**82.72**	**81.98**
Sesquilignans ^3^	9.74	9.40	9.07	10.14
Dilignans ^3^	5.84	5.23	5.07	5.24
**Sum of sesqui/dilignans**	**15.57**	**14.63**	**14.14**	**15.38**
**Sum of non-identified**	**2.83**	**2.50**	**2.97**	**2.39**
**Total exractives**	**100.00**	**100.00**	**100.00**	**100.00**

^1^ Additional knot of the same whorl, which was chosen for STXM. ^2^ Knot that was used for STXM analysis (cryosectioned sample). ^3^ Analysed by a short column.

**Table 4:** Element composition of the resin-embedded tracheid regions measured with STEM-EDS (Figure 7).

	Atom–%			Mass–%		
	C	O	Os	C	O	Os
1	92.55	7.42	0.03	89.94 ± 0.57	9.60 ± 0.22	0.45 ± 0.08
2	91.38	8.00	0.62	81.69 ± 0.79	9.53 ± 0.31	8.79 ± 0.46
3	95.39	4.59	0.02	93.73 ± 0.76	6.01 ± 0.22	0.26 ± 0.10

**Table 5:** Element composition of the cell wall regions measured with STEM-EDS (Figure 8).

	Atom–%			Mass–%		
	C	O	Os	C	O	Os
**1**	91.68	8.26	0.06	88.44 ± 0.75	10.62 ± 0.30	0.94 ± 0.16
**2**	92.73	7.25	0.02	90.30 ± 1.15	9.41 ± 0.43	0.29 ± 0.22
**3**	93.42	6.54	0.04	90.92 ± 0.61	8.48 ± 0.22	0.60 ± 0.10
**4**	94.61	5.33	0.06	92.11 ± 0.71	6.90 ± 0.22	0.98 ± 0.06
**5**	95.54	5.38	0.08	91.83 ± 0.50	6.97 ± 0.16	1.20 ± 0.08

## Supplementary Materials

The following are available online at https://www.mdpi.com/1420-3049/25/13/2997/s1, Figure S1: Reference O1s XAS of milled wood lignin, the tissue culture lignin, HMR, and the acetone extract, Figure S2: STXM analysis of a Norway spruce cryosection showing a dehydroabietic acid-like deposit.

## Sample Availability

STXM and XAS data are available at http://urn.fi/urn:nbn:fi:att:e85f4e20-40a7-4350-b0b9-a0f4fcc716ce.
