# Supplementary material for: Lignans in Knotwood of Norway Spruce: Localisation with Soft X-ray Microscopy and Scanning Transmission Electron Microscopy with Energy Dispersive X-ray Spectroscopy
Source: Molecules. 2020 Jun 30;25(13):2997. doi: 10.3390/molecules25132997 (PMC7411943; doi:10.3390/molecules25132997)
Supplement: Supplementary file 1 [file molecules-25-02997-s001.pdf]

Supplementary Material for Lignans in Knotwood of Norway Spruce: Localisation with Soft X-Ray Microscopy and Scanning Transmission Electron Microscopy with Energy Dispersive X-Ray Spectroscopy

Tuomas Mansikkala [1,2], Minna Patanen [1,2,*], Anna Kärkönen [3], Risto Korpinen [3], Andrey Pranovich [4], Takuji Ohigashi[5], Sufal Swaraj[6], Jani Seitsonen[7], Janne Ruokolainen[7], Marko Huttula[1], Pekka Saranpää[3], and Riikka Piispanen [3,*]

1 Nano and Molecular Systems Research Unit, Faculty of Science, University of Oulu, P.O. Box 8000, FI-90014 University of Oulu, Finland; leo.mansikkala@oulu.fi (T.M.); marko.huttula@oulu.fi (M.H.)
2 Biocenter Oulu, P.O. Box 5000, FI-90014 University of Oulu, Finland;
3 Production Systems, Natural Resources Institute Finland, Latokartanonkaari 9, FI-00790 Helsinki, Finland; anna.karkonen@luke.fi (A.K.); risto.korpinen@luke.fi (R.K.); pekka.saranpaa@luke.fi (P.S.)
4 Wood and Paper Chemistry Research Group, Laboratory of Natural Materials Technology, Åbo Akademi University, Porthansgatan 3, FI-20500 Turku/Åbo, Finland; andrey.pranovich@abo.fi (A.P.)
5 UVSOR facility, Institute for Molecular Science, 38 Nishigo-naka, Myodaiji, Okazaki, Aichi 444-8585, Japan; ohigashi@ims.ac.jp (T.O.)
6 SOLEIL Synchrotron, L'Orme des Merisiers, Saint-Aubin, P.O. Box 48, FR-91192 Gif-Sur-Yvette Cedex, France; sufal.swaraj@synchrotron-soleil.fr (S.S.)
7 Nanomicroscopy Center, Department of Applied Physics, Aalto University, P.O. Box, 15100, FI-00076 AALTO, Finland; jani.seitsonen@aalto.fi (J.S.); janne.ruokolainen@aalto.fi (J.R.)
*Correspondence: riikka.piispanen@luke.fi (R.P.); Tel.: +358-29-532-5473; minna.patanen@oulu.fi (M.P.); Tel.: +358-29-448-1326

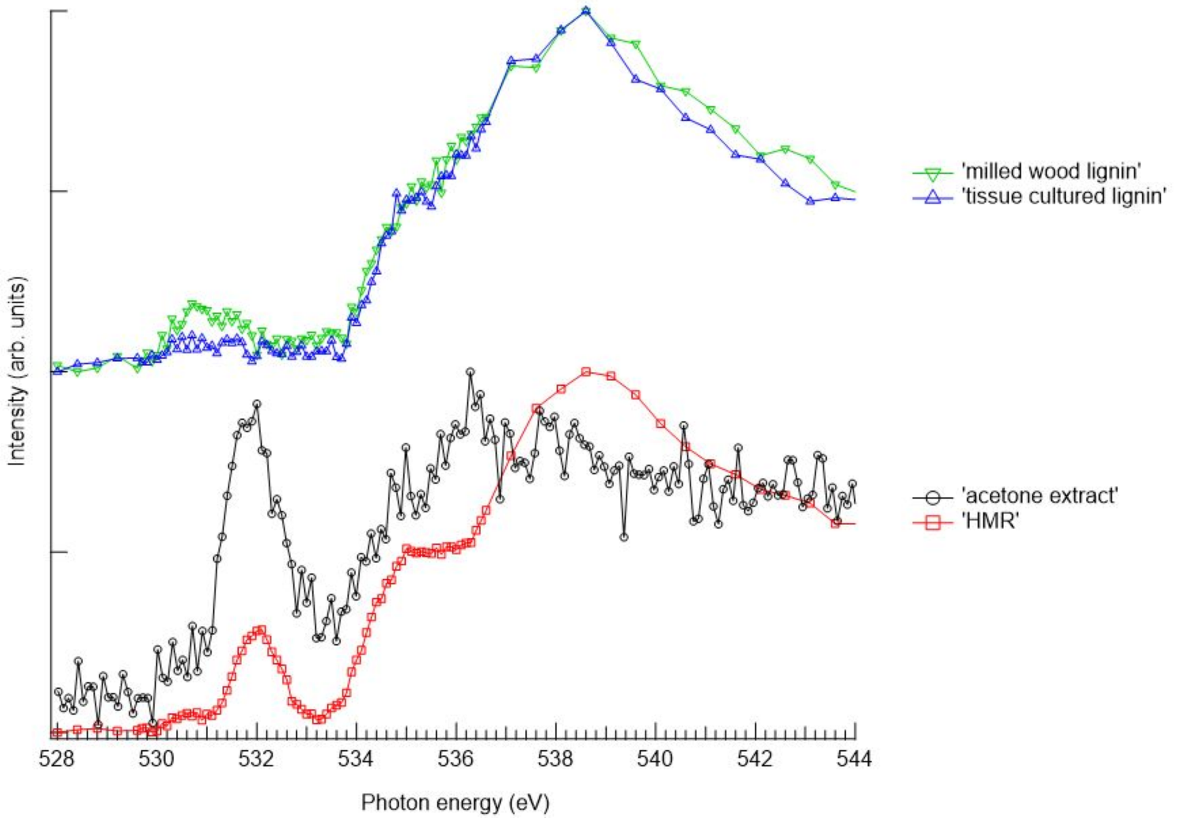

Figure S1. O 1s X-ray absorption spectra of the acetone extract of the knotwood (black line with circles), of hydroxymatairesinol (HMR, red line with squares), of the tissue culture lignin (blue line with triangles), milled wood lignin (green line with triangles). The spectra of HMR and the tissue culture lignin have been adopted from Huttula *et al*. (Huttula 2018).

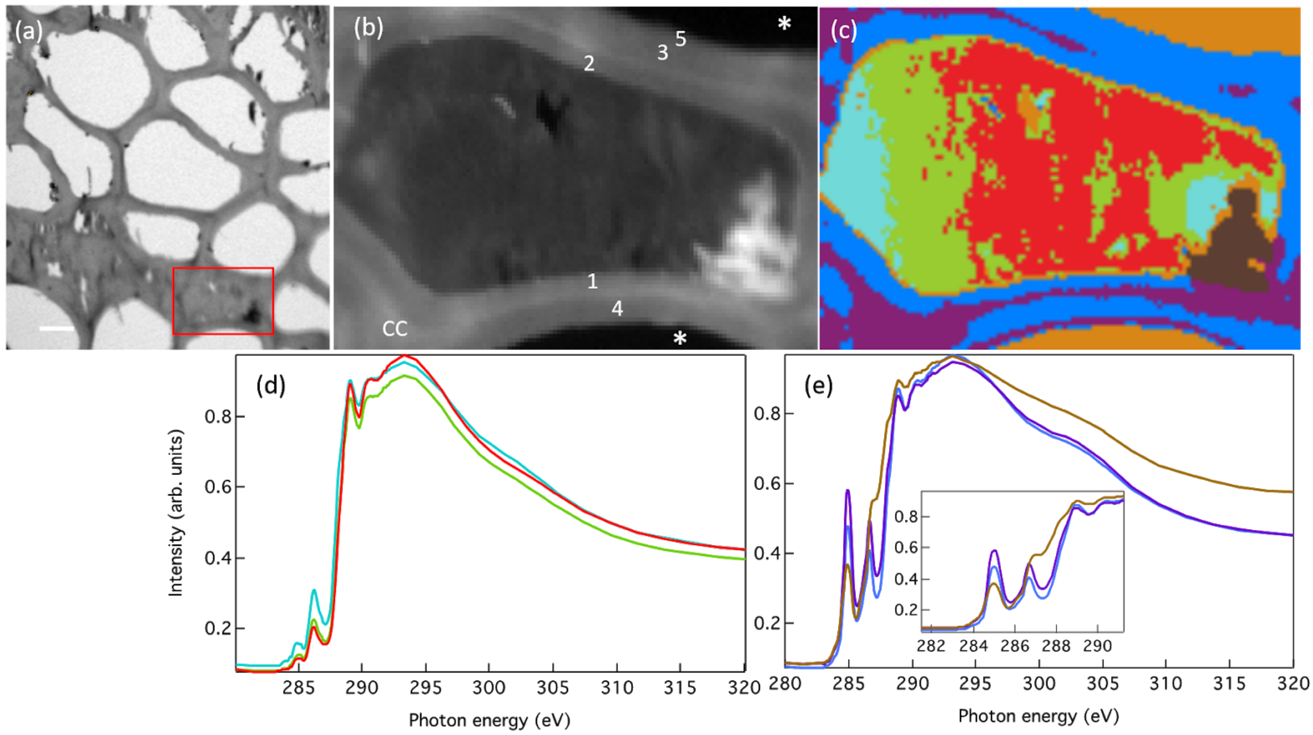

Figure S2. (a) Cryosectioned Norway Spruce knotwood imaged at 350 eV photon energy. **(b)** Average image of energy range 284.0-287.5 eV of the ray tracheid/epithelial cell marked with a red rectangle in (a). CC and numbers 1-5 indicate the approximate regions from which the representative spectra for cell corner and compound middle lamella and cell wall regions were extracted. Asterisk (*) indicate the axial tracheids surrounding the ray tracheid. **(c)** Cluster analysis of (b). **(d)** C 1s XAS assigned to red, green, and teal clusters drawn with corresponding colors. **(e)** C 1s XAS assigned to violet, blue, and brown clusters drawn with corresponding colors. The inset shows the fingerprint region for aromatic compounds enlarged.

An STXM measurement at C 1s edge of a cryosectioned ray tracheid or a resin duct epithelial cell surrounded by axial tracheids. A cutting surface of a longitudinal cell wall revealed a small deposit with a dehydroabietic acid-type spectrum (Figure S2e). The spectral analysis shows that the longitudinally cut wall is only slightly lignified (Figure S2d), but contains hemicellulose/cellulose-like spectra (Huttula et al. 2018). Instead, the surrounding transversely cut cell walls were lignified. The S2 layer of the ray tracheid/epithelial cell is thinner than that of the axial tracheid. C 1s XAS spectral fitting revealed that the lignin content in the thinner wall is approximately 40-45 % (regions where spectra were extracted are marked with 1 and 2 in Figure S2b). In the cell walls of axial tracheids, three regions were selected for the fitting. The ones closer to the compound middle lamella (3 and 4 in Figure S2b) gave an approximate lignin content of 47-49 %, whereas region 5 close to the axial tracheid lumen gave lignin content of approximately 27-32 %.
